# Unpacking reappraisal: a systematic review of fMRI studies of distancing and reinterpretation

**DOI:** 10.1093/scan/nsad050

**Published:** 2023-09-27

**Authors:** Bryan T Denny, Mallory L Jungles, Pauline N Goodson, Eva E Dicker, Julia Chavez, Jenna S Jones, Richard B Lopez

**Affiliations:** Department of Psychological Sciences, Rice University, Houston, TX 77005, USA; Department of Psychological Sciences, Rice University, Houston, TX 77005, USA; Department of Psychological Sciences, Rice University, Houston, TX 77005, USA; Department of Psychology, Seattle University, Seattle, WA 98122, USA; Department of Psychological Sciences, Rice University, Houston, TX 77005, USA; Department of Psychological Sciences, Rice University, Houston, TX 77005, USA; Department of Psychological & Cognitive Sciences, Worcester Polytechnic Institute, Worcester, MA 01609, USA

**Keywords:** emotion regulation, reappraisal, psychological distancing, reinterpretation, meta-analysis

## Abstract

**In recent decades, a substantial volume of work has examined the neural mechanisms of cognitive reappraisal. Distancing and reinterpretation are two frequently used tactics through which reappraisal can be implemented. Theoretical frameworks and prior evidence have suggested that the specific tactic through which one employs reappraisal entails differential neural and psychological mechanisms. Thus, we were motivated to assess the neural mechanisms of this distinction by examining the overlap and differentiation exhibited by the neural correlates of distancing (specifically via objective appraisal) and reinterpretation. We analyzed 32 published** functional magnetic resonance imaging (**fMRI) studies in healthy adults using multilevel kernel density analysis. Results showed that distancing relative to reinterpretation uniquely recruited right** bilateral dorsolateral PFC (**DLPFC) and left posterior parietal cortex, previously associated with mentalizing, selective attention and working memory. Reinterpretation relative to distancing uniquely recruited left** bilateral ventrolateral PFC (**VLPFC), previously associated with response selection and inhibition. Further, distancing relative to reinterpretation was associated with greater prevalence of bilateral amygdala attenuation during reappraisal. Finally, a behavioral meta-analysis showed efficacy for both reappraisal tactics. These results are consistent with prior theoretical models for the functional neural architecture of reappraisal via distancing and reinterpretation and suggest potential future applications in region-of-interest specification and neural network analysis in studies focusing on specific reappraisal tactics.**

In the past two decades, a significant and growing number of studies have examined the neural mechanisms of cognitive reappraisal, an emotion regulation strategy that involves changing one’s construal of an emotion-eliciting stimulus in a way that alters its emotional impact (Gross, 1998, 2015). While importantly the adaptiveness and success of any emotion regulation strategy are ultimately driven by factors related to the person and situation involved in addition to the strategy utilized (Dore *et al.*, 2016), reappraisal has frequently been shown to be adaptive in a variety of contexts and when compared to other regulation strategies, like expressive suppression (Gross and John, 2003; Webb *et al.*, 2012; Gross, 2015). Further, reappraisal represents an essential component of many forms of psychotherapy, including cognitive behavioral therapy (Berking *et al.*, 2008; Beck, 2011). As such, there has been considerable interest in investigating the neural mechanisms through which reappraisal occurs (Kalisch, 2009; Diekhof *et al.*, 2011; Buhle *et al.*, 2014; Kohn *et al.*, 2014; Messina *et al.*, 2015; Morawetz *et al.*, 2017; Picó-Pérez *et al.*, 2017).

While many studies have examined reappraisal relative to unregulated baseline conditions or via comparison to distinct emotion regulation strategies, reappraisal can be operationalized via a broad and heterogeneous array of cognitive change tactics that may be implemented alone or in tandem (McRae *et al.*, 2012). Two such commonly employed tactics of particular interest when using reappraisal to down-regulate negative emotion are psychological distancing and reinterpretation (Denny and Ochsner, 2014; Denny, 2020). Psychological distancing may be operationalized as appraising an emotional stimulus as an objective, impartial observer or by increasing the perceived spatial or temporal distance from an emotionally evocative stimulus in ways described by construal level theory (Trope and Liberman, 2010; Powers and LaBar, 2019; Shahane *et al.*, 2021). In the current work, we were particularly interested to target distancing studies that operationalized distancing using the objective, impartial appraisal tactic, given the need to parse facets of distancing (Powers and LaBar, 2019; Denny, 2020) and given interest in the objective, impartial form in particular (Shahane and Denny, 2019; Shahane *et al.*, 2021). For example, if you’re experiencing a negative situation you can’t easily change fundamentally, like anticipating a high-stakes exam about which you’re feeling anxious, a distanced perspective may lead you to realize that taking exams is a natural and understandable aspect of assessment in a course.

Alternatively, you may engage in reinterpretation, which is frequently operationalized by imagining that a situation isn’t as bad as it first seemed, by imagining that the situation will improve with time, or by questioning the reality of the situation or stimulus (Silvers *et al.*, 2015). While questioning the reality of a stimulus is sometimes referred to as hypothetical distancing (Trope and Liberman, 2010), it is often operationalized as reinterpretation (Burklund *et al.*, 2014; Silvers *et al.*, 2015). Thus, in the previous example regarding the exam, when reinterpreting you may imagine that despite the anxiety you are going to do quite well on the exam, and in any case it’s not a make-or-break situation.

Several recent neuroimaging meta-analyses have examined the neural mechanisms of reappraisal as a whole in healthy adult populations, collapsing across differential means through which reappraisal can be operationalized including distancing and reinterpretation. These meta-analyses have broadly converged in showing recruitment of domain-general cognitive control regions including dorsomedial prefrontal cortex (DMPFC), bilateral dorsolateral PFC (DLPFC), bilateral ventrolateral PFC (VLPFC) and bilateral posterior parietal cortex in conjunction with recruitment of left posterior temporal cortex, associated with semantic and perceptual representations, during reappraisal implementation in healthy adults (Kalisch, 2009; Diekhof *et al.*, 2011; Buhle *et al.*, 2014; Kohn *et al.*, 2014; Messina *et al.*, 2015; Morawetz *et al.*, 2017).

The results of these prior meta-analyses are largely consistent with a model whereby reappraisal involves the recruitment of domain-general cognitive control regions in DMPFC, DLPFC, VLPFC and posterior parietal cortex that modulate semantic representations in left posterior temporal cortex in ways that indirectly modify emotional responses (Buhle *et al.*, 2014). DMPFC has been consistently implicated in mentalizing about oneself and others (Denny *et al.*, 2012; Ochsner *et al.*, 2012; Powers and LaBar, 2019). DLPFC, posterior DMPFC near Brodmann area 8 and posterior parietal cortex have been associated with selective attention and maintenance of information in working memory (Miller, 2000; Wager and Smith, 2003; Wager *et al.*, 2004; Owen *et al.*, 2005; Ochsner *et al.*, 2012; Powers and LaBar, 2019). Further, posterior parietal cortex in particular, including the angular gyrus and temporoparietal junction (TPJ), has additionally been associated with convergent mentalizing, attention, language and memory processing (Ochsner *et al.*, 2012; Carter and Huettel, 2013; Powers and LaBar, 2019). In addition, VLPFC has been especially implicated in the selection of goal appropriate responses and the inhibition of goal-inappropriate responses (Aron *et al.*, 2004, 2014; Badre *et al.*, 2005). Thus, drawing on these cognitive control functions, models for reappraisal have described the involvement of DMPFC in reflecting on and monitoring changing mental states, DLPFC and posterior parietal cortex in holding reappraisals in mind, and VLPFC in selecting appropriate reappraisals, which serve to modulate activity in regions associated with emotion generation, including the insula, ventral striatum and particularly the amygdala (Ochsner and Gross, 2005, 2008; Ochsner *et al.*, 2012; Buhle *et al.*, 2014; Denny and Ochsner, 2018).

Notably, Buhle and colleagues did not find consistent evidence of reappraisal-related recruitment of ventromedial PFC (VMPFC), previously shown to be essential for fear extinction (Milad and Quirk, 2012); this result suggests that, while both reappraisal and fear extinction entail modulation of an emotional response, reappraisal engages divergent psychological and neural mechanisms that may involve constructing alternative internal representations in ways supported by left posterior temporal cortex (Ochsner and Gross, 2005, 2007; Vigneau *et al.*, 2006; Van Overwalle and Baetens, 2009; Ochsner *et al.*, 2012; Buhle *et al.*, 2014). Other meta-analyses have found results largely consistent to that of Buhle and colleagues (Kalisch, 2009; Diekhof *et al*., 2011; Kohn *et al.*, 2014; Messina *et al.*, 2015; Morawetz *et al.*, 2017) did report a prominent role for VMPFC in reappraisal implementation (Diekhof *et al.*, 2011). We were thus motivated to assess the extent of VMPFC involvement when examining reappraisal tactics separately as well as jointly.

Indeed, prior psychological models for reappraisal and prior work have suggested that the specific tactic through which one employs reappraisal may have important differential consequences for behavior and experience (McRae *et al.*, 2012; Webb *et al.*, 2012; Denny and Ochsner, 2014; Messina *et al.*, 2015; Denny, 2020). Webb and colleagues highlighted the need for a taxonomy of reappraisal tactics, with a key distinction being reappraisal tactics that operate via changing one’s construal of the situation (e.g. reinterpretation) *vs* reappraisal tactics that operate via taking another perspective (e.g. an objective and impartial perspective via psychological distancing); this distinction was also made by (Ochsner *et al*., 2004) in one of three fMRI studies to our knowledge that have directly compared the neural correlates of reinterpretation and distancing in the same study. Ochsner and colleagues found that implementing distancing (referred to as self-focused reappraisal) *vs* reinterpretation when downregulating negative emotion was associated with medial PFC and left posterior parietal cortex activity, whereas implementing reinterpretation (referred to as situation-focused reappraisal) *vs* distancing was associated with bilateral DLPFC, VLPFC and left posterior temporal cortex activity (Ochsner *et al.*, 2004). Dörfel and colleagues further found that distancing recruited posterior parietal cortex/angular gyrus relative to reinterpretation, whereas reinterpretation selectively recruited a diverse set of lateral frontal, temporal and occipital areas relative to distancing (D̈rfel *et al.*, 2014). A third recent study found no significant results for a contrast of distancing *vs* reinterpretation, but found right anterior temporal gyrus activity as well as increased amygdala and insula activity for a contrast of reinterpretation *vs* distancing (Hermann *et al.*, 2021). Further, there is behavioral evidence that distancing relative to reinterpretation involves distinct psychological mechanisms and training profiles, with longitudinal training in distancing uniquely leading to longitudinal reductions in perceived stress (Denny and Ochsner, 2014), which may be due, in part, to greater stimulus independence and cognitive automatability for distancing relative to reinterpretation (Denny and Ochsner, 2014; Denny, 2020).

Two prior meta-analyses have directly compared the neural correlates of distancing and reinterpretation implementation (Messina *et al.*, 2015; Picó-Pérez *et al.*, 2017), each using relatively modest study databases. Messina and colleagues, in a meta-analysis of seven studies involving distancing and eight studies involving reinterpretation, found only limited evidence for differentiation between tactics in a direct comparison, reporting a single cluster specific to reinterpretation relative to distancing in left supplementary motor area/Brodmann area 6, and no clusters for distancing *vs* reinterpretation. While Picó-Pérez and colleagues primarily examined differences in neural correlates between patients with mood and anxiety disorders relative to healthy adults, the authors performed an exploratory meta-analysis with three studies specific to distancing and five studies specific to reinterpretation that each contained healthy and clinical samples. The authors found that, relative to patients, healthy adults recruited a diverse set of regions including bilateral angular gyrus, bilateral DLPFC, VMPFC and left posterior temporal cortex for a contrast of distancing *vs* reinterpretation. Conversely, in healthy adults relative to patients, reinterpretation relative to distancing was associated with only left VLPFC and left temporal gyrus activity (Picó-Pérez *et al.*, 2017).

Thus, the neurocognitive model for reappraisal as a whole described above may be differentially applicable, or applicable to differing degrees or weights, as a function of the reappraisal tactic employed. We were therefore motivated to perform a quantitative meta-analysis to assess the degree of overlap and differentiation between the neural correlates of distancing and reinterpretation implementation in healthy adults using a larger study database than has been previously applied to this question. We predicted that distancing and reinterpretation would have significant overlap in neural correlates consistent with the neurocognitive model for reappraisal above. However, we also predicted differentiation as a function of tactic, with distancing expected to engage greater activity in regions associated with mentalizing and perspective taking, including DMPFC and left posterior parietal cortex. Reinterpretation relative to distancing, in contrast, was expected to engage greater activity in regions associated with response selection and inhibition, including VLPFC. Finally, we also performed an exploratory behavioral meta-analysis on available self-reported data from the included studies to further characterize similarities and differences between distancing and reinterpretation.

## Methods

This systematic review was performed according to the checklist of Preferred Reporting Items for Systematic Review and Meta Analyses (http://prisma-statement.org/prismastatement/Checklist.aspx; see Supplementary Material for checklist).

### Identification of studies

Studies were initially identified and abstracts were screened by a trained researcher through keyword searches of PubMed, Google Scholar and OneSearch (on library.rice.edu). Broad database search terms included ‘emotion’, ‘emotion regulation’, ‘reappraisal’, ‘fMRI’, ‘amygdala’, ‘cognitive’ and ‘prefrontal cortex’ for papers published between 2001 and 2019. Simultaneously, papers that were cited in meta-analyses conducted by Buhle *et al.* (2014) and Powers and LaBar (2019) were reviewed. Authors of studies that did not include publicly available results tables of interest were contacted with requests to provide results tables for these contrasts.

### Eligibility criteria

The following inclusion criteria were used:

1. Study reports results from a task-based reappraisal experiment involving down-regulation of negative emotion in healthy adults.
2. Study used functional magnetic resonance imaging (fMRI).
3. Study contained at least one contrast that compared either distancing or reinterpretation (separately) to an unregulated baseline condition (e.g. ‘Look’).

The following exclusion criteria were used:

1. Study did not include healthy adults.
2. Study did not use negatively valanced visual stimuli.
3. Study did not include a reappraisal task completed within an fMRI scanner.
4. Study included an intervention aside from reappraisal training.
5. Study did not include a whole-brain contrast specific to distancing or reinterpretation in comparison to an unregulated baseline (e.g. ‘Look’) condition. Contrasts were also excluded if contrasts directly compared distancing to reinterpretation or vice versa.
6. Study conducted only multivariate instead of univariate analyses.

### Study coding and data collection

A total of four trained coders from Rice University independently reviewed each study and documented detailed participant and intervention characteristics, including the study population, the total number of healthy adult participants and the type and valence of the stimulus used in the emotion regulation task. The coders also reviewed the type of reappraisal strategy used in the study. Reappraisal strategies were coded as reinterpretation or distancing according to the degree to which the instructions given to participants matched with pre-determined operational definitions of ‘distancing’ and ‘reinterpretation’. We considered a study to pertain to distancing if participants were instructed to adopt a detached or objective perspective when viewing negative images or clips in the emotion regulation task. We considered a study to pertain to reinterpretation if participants were instructed to tell themselves a story about the negative image or clip that would make the situation at hand seem less negative than it may initially have seemed. Disagreements were resolved via consensus. Coders also reviewed the type of whole-brain contrast conducted in each study (e.g. ‘Distancing > Look Negative’ or ‘Reinterpretation > Look Negative’). In addition to the type of contrast, coders reviewed the direction of the contrast (e.g. ‘Reappraise Negative > Look Negative’ or ‘Look Negative > Reappraise Negative’), as well as the stereotaxic coordinate space in which results were reported. All multiple comparison corrected results from individual studies, excluding local maxima and including small volume corrected results, were included.

As shown in Table 1, following application of inclusion and exclusion criteria, 16 distancing contrasts and 17 reinterpretation contrasts were included in the present meta-analysis. Risk of bias was low (see quality and heterogeneity assessment methods in Supplemental Materials).

**Table 1.** Included study characteristics

	Healthy sample		
Author	*N* (female)	Cognitive reappraisal strategy used	Stimulus material
Burklund *et al.*, 2014	39 (31)	Reinterpretation	Negative images (IAPS)
Denny *et al.*, 2015a	21 (11)	Distancing	Negative images (IAPS)
Denny *et al.*, 2015b	17 (12)	Distancing	Negative images (IAPS)
D̈rfel *et al.*, 2014	17 (17); 19 (19)	Distancing; Reinterpretation	Negative images (IAPS)
Erk *et al.*, 2010	17 (8)	Distancing	Negative images (IAPS)
Gianaros *et al.*, 2014	157 (80)	Reinterpretation	Negative images (IAPS)
Goldin *et al.*, 2008	17 (17)	Distancing	15 sec film clips of disgust-inducing surgical procedures, vomiting, and animal slaughter
Hallam *et al.*, 2015	20 (10)	Distancing	Sad or disgusting images (IAPS)
Hayes *et al.*, 2010	25 (11)	Distancing	Negative images (IAPS)
Koenigsberg *et al.*, 2009	16 (9)	Distancing	Negative images (IAPS)
McRae *et al.*, 2010	18 (18)	Reinterpretation	Negative images (IAPS)
Modinos *et al.*, 2010	18 (7)	Reinterpretation	Negative images (IAPS)
Nelson *et al.*, 2015	21 (11)	Reinterpretation	Negative images of faces (IAPS)
New *et al.*, 2009	14 (14)	Reinterpretation	Negative images (IAPS)
Opitz *et al.*, 2012	31 (17)	Reinterpretation	Negative images (IAPS)
Paschke *et al.*, 2016	108 (55)	Distancing	Negative images
Qu and Telzer, 2017	29 (14)	Reinterpretation	Images of European or Asian individuals in an emotionally negative situation
Reinecke *et al.*, 2015	18 (14)	Reinterpretation	Negative images (IAPS)
Schardt *et al.*, 2010	37 (37)	Distancing	Negative images (IAPS)
Schulze *et al.*, 2011	15 (15)	Distancing	Negative images (IAPS)
Seidel *et al.*, 2018	36 (36)	Distancing	Negative images
Shermohammed *et al.*, 2017	54 (27)	Reinterpretation	Negative images (IAPS)
Silvers *et al.*, 2015	30 (13)	Reinterpretation	Negative images
Simsek *et al.*, 2017	15 (15)	Reinterpretation	Negative images (IAPS)
Sripada *et al.*, 2014	49 (23)	Reinterpretation	Negative images (IAPS)
Townsend *et al.*, 2013	26 (11)	Reinterpretation	Negative images (IAPS)
Vanderhasselt *et al.*, 2013	42 (42)	Reinterpretation	Negative images (IAPS)
Walter *et al.*, 2009	18 (18)	Distancing	Negative images (IAPS)
Winecoff *et al.*, 2011	42 (21)	Distancing	Negative images (IAPS)
Winecoff *et al.*, 2013	31 (21)	Distancing	Negative images (IAPS)
Xie *et al.*, 2016	19 (19)	Distancing	Negative images (IAPS)
Ziv *et al.*, 2013	27 (13)	Reinterpretation	Negative images (IAPS)

### Data analysis overview

In order to address our questions regarding the overlap and differentiation of neural correlates pertaining to psychological distancing and reinterpretation, we took a comprehensive approach as follows. To examine consistency with the existing literature on reappraisal as a whole, we first performed a meta-analysis for a combination of distancing and reinterpretation *vs* an unregulated baseline (e.g. ‘Look’) as described below. We next performed meta-analyses for distancing and reinterpretation separately, followed by a conjunction analysis for which brain regions were present in both of the individual meta-analyses for distancing and reinterpretation. Then, we performed a meta-analysis directly comparing distancing and reinterpretation to each other. Finally, we performed a meta-analysis for the reverse contrast reflective of attenuation due to reappraisal (Look Negative > Reappraise Negative using either distancing or reinterpretation).

### Multilevel kernel density analysis

To perform the meta-analyses above, we used multilevel kernel density analysis (MKDA; Wager *et al.*, 2007). This approach treats contrasts as the unit of analysis. Coordinates reported in Talairach space were transformed to Montreal Neurological Institute (MNI) space using the tal2mni algorithm (Matthew Brett; https://www.sdmproject.com/utilities/). We then assessed the spatial density of reported peaks using MKDA. As described previously, this approach nests peaks within contrasts, thus controlling for the total number of peaks a given contrast reports (Wager *et al.*, 2007; Kober *et al.*, 2008; Denny *et al.*, 2012; Buhle *et al.*, 2014).

Next, a 12 mm radius Gaussian smoothing kernel was created around each peak, whereby voxels closest to the peak were given a value of 1, with those further away receiving values closer to 0. From this, we created graded contrast-indicator maps (CIMs) for each peak reported. These CIMs were then combined to form a single nested map for each contrast. As such, each contrast could contribute no more than a value of 1 for a given voxel when the overall proportion statistics were computed. Combined CIMs were then weighted by the square root of the sample size of the contrast; thus, contrasts containing relatively many participants exerted greater weight on the results, and contrasts containing relatively few participants were down-weighted.

Statistical inferences were made by comparing the proportion of contrasts showing activation in a given voxel to an empirical null distribution derived by Monte Carlo random sampling of spatially scrambled peaks (Wager *et al.*, 2007; Kober *et al.*, 2008; Denny *et al.*, 2012; Buhle *et al.*, 2014). Data were resampled to 3 mm isotropic voxels. Each Monte Carlo simulation consisted of 5000 iterations. Results were masked using a gray matter mask created through segmentation of the MNI-T1 template (the ‘Colin’ brain) (45,557 voxels in 3 mm isotropic resolution).

All analyses except for the conjunction analysis were thresholded to control for the family-wise error (FWE) rate using voxel-wise whole-brain correction. That is, individual voxels were thresholded according to the distribution of all observed maximum values from the Monte Carlo simulations; a given voxel was significant where the proportion statistic for that voxel exceeded the 95th percentile of all observed maximum values during Monte Carlo simulations (i.e. *P* < 0.05, whole-brain corrected). For the conjunction analysis of individual meta-analyses for distancing and reinterpretation for the Reappraise Negative > Look Negative contrast, results were FWE-corrected using thresholds determined via alphasim (Ward, 2000) using 5000 Monte Carlo iterations for a conjunction of two independent maps, resulting in a threshold of *P* < 0.001, two-tailed, uncorrected; extent threshold = 2 voxels; *P* < 0.05, two-tailed, FWE-corrected. All analyses were performed using NeuroElf v1.1 (neuroelf.net). Reported coordinates are in MNI space.

### Behavioral meta-analysis

Finally, to compare distancing and reinterpretation utilizing self-reported affect data from the included studies, we conducted an exploratory behavioral meta-analysis (see Supplemental Material).

## Results

### Study selection and study characteristics

**Fig. 1.**
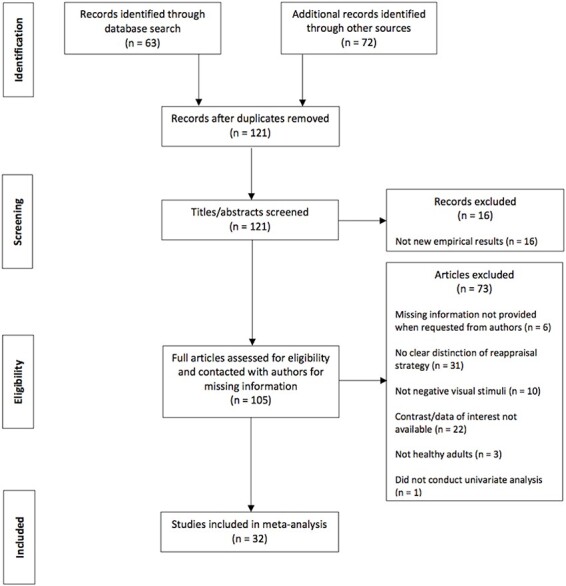
PRISMA Flow Diagram (http://prisma-statement.org/prismastatement/flowdiagram.aspx).

A total of 135 potentially relevant records were identified through database searches. After removing duplicates, abstracts of the 121 remaining articles were screened, and 105 advanced to the coding process for further review. Finally, after contacting authors for missing information and assessing the remaining articles for eligibility, we included 32 articles in our meta-analysis. The basis of exclusion for potentially relevant records varied from not conducting univariate analyses, not including healthy adults, etc. The study selection process is shown in Figure 1.

Detailed characteristics of the included studies are documented in Table 1. All study populations included healthy adults, with sample sizes ranging from 14 to 157 individuals, for a total of 1,063 participants overall.

### Reappraise Negative > Look Negative

Significant cluster results for the contrast Reappraise Negative > Look Negative can be found in Table 2. To address our questions, we first performed a meta-analysis collapsing across distancing and reinterpretation (Table 2). Consistent with prior meta-analyses on reappraisal as a whole, this analysis yielded activation of bilateral DLPFC, left VLPFC, DMPFC, posterior DMPFC/pre-supplementary motor area (pre-SMA), left posterior temporal cortex and posterior cingulate cortex.

**Table 2.** Results of meta-analysis for contrast Reappraise Negative > Look Negative

Comparison	Region	MNI coordinates (x, y, z)	Number of voxels	*z* (peak)	Max proportion differential	Mean proportion differential
Distancing + Reinterpretation (combined)	Left posterior DLPFC/premotor cortex (BA 6)	−42, 9, 48	22	3.72	0.178285	0.148885
	Left posterior DMPFC/pre-SMA (BA 6)	−9, 12, 63	31	3.72	0.173159	0.147754
	Left VLPFC (BA 47)	−48, 30, −9	21	3.72	0.169375	0.144415
	Right DLPFC (BA 9)	39, 21, 42	8	3.72	0.150306	0.142219
	Left posterior temporal cortex (BA 22)	−60, −36, −3	9	3.72	0.143010	0.133850
	Left DMPFC (BA 8)	−3, 27, 51	4	3.72	0.136380	0.132294
	Posterior cingulate cortex (BA 23)	0, −21, 33	1	3.72	0.133870	0.133870
Distancing > Null	Right DLPFC (BA 8)	39, 21, 45	30	3.72	0.296965	0.237250
	Left posterior parietal cortex (BA 40)	−54, −54, 45	12	3.72	0.235314	0.218557
	Posterior cingulate cortex (BA 23)	0, −24, 30	4	3.72	0.220304	0.210516
	Right posterior parietal cortex (BA 40)	57, −51, 36	6	3.72	0.211593	0.205035
	Left posterior DMPFC/pre-SMA (BA 6)	−9, 15, 60	3	3.72	0.204325	0.203677
Reinterpretation > Null	Left VLPFC (BA 47)	−48, 30, −9	17	3.72	0.269161	0.230847
	Left posterior DLPFC/premotor cortex (BA 6)	−42, 6, 48	12	3.72	0.257754	0.224491
	Left posterior DMPFC/pre-SMA (BA 6)	−6, 9, 66	1	3.72	0.209440	0.209440
Distancing + Reinterpretation (conjunction)	Left posterior DMPFC/pre-SMA (BA 6)	−6, 12, 60	24	3.72	0.165728	0.129026
	Left posterior temporal cortex (BA 22)	−60, −36, −3	17	3.35	0.133843	0.121380
	Left posterior DLPFC/premotor cortex (BA 6)	−42, 12, 48	7	3.54	0.130479	0.116664
	Left DMPFC (BA 8)	−3, 24, 51	5	3.16	0.125881	0.115333
Distancing > Reinterpretation	Right DLPFC (BA 8)	39, 21, 45	26	3.72	0.281653	0.226832
	Left posterior parietal cortex (BA 40)	−54, −54, 45	10	3.72	0.225482	0.208517
Reinterpretation > Distancing	Left VLPFC (BA 47)	−48, 30, −9	6	3.72	0.216788	0.203978

BA = Brodmann Area. Proportion Differential = Difference in activation proportion between conditions in brain region.

**Fig. 2.**
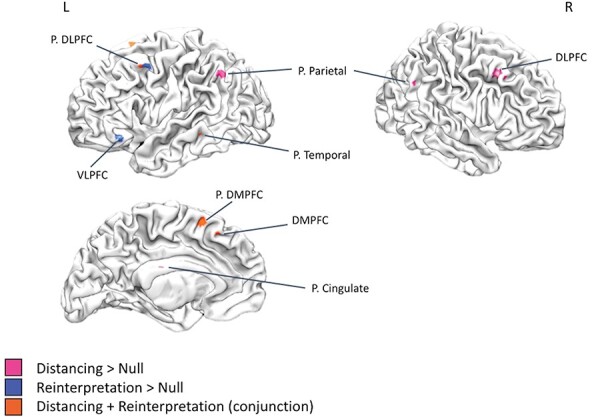
Meta-analysis results for distancing, reinterpretation and the conjunction of distancing and reinterpretation for the Reappraise Negative > Look Negative contrast. P. = posterior. DLPFC = dorsolateral PFC. DMPFC = dorsomedial PFC. VLPFC = ventrolateral PFC.

Next, we performed meta-analyses for distancing and reinterpretation separately (Figure 2; Table 2). For distancing, we observed consistent activation in right DLPFC, posterior DMPFC/pre-SMA, bilateral posterior parietal cortex and posterior cingulate cortex. For reinterpretation, we observed consistent activation in left VLPFC, left posterior DMPFC/pre-SMA and left posterior DLPFC/premotor cortex.

We next performed a conjunction of the individual meta-analytic maps for distancing and reinterpretation to assess overlap between them (Figure 2; Table 2). We observed common activation in left posterior DMPFC/pre-SMA, DMPFC, left posterior temporal cortex and left posterior DLPFC/premotor cortex.

**Fig. 3.**
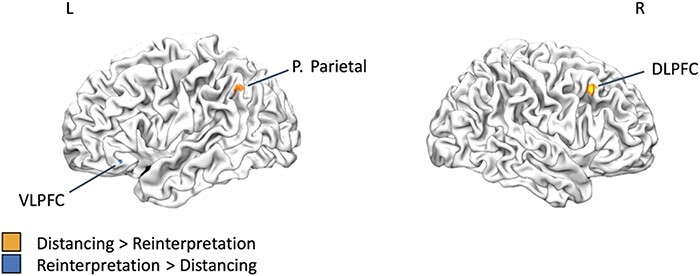
Direct comparison of distancing and reinterpretation for the Reappraise Negative > Look Negative contrast. P. = posterior. DLPFC = dorsolateral PFC. VLPFC = ventrolateral PFC.

Finally, we performed a meta-analysis to directly contrast the neural correlates of distancing and reinterpretation. Implementing distancing resulted in significantly more consistent activation in right DLPFC and left posterior parietal cortex relative to reinterpretation, whereas implementing reinterpretation resulted in significantly more consistent activation in left VLPFC relative to distancing (Figure 3; Table 2).

### Look Negative > Reappraise Negative

We also performed a meta-analysis for the reverse contrast, Look Negative > Reappraise Negative. This contrast reflects regions that have shown significant attenuation in activity due to reappraisal. Significant cluster results for the contrast Look Negative > Reappraise Negative are shown in Table 3. When collapsing across distancing and reinterpretation, as expected, extensive bilateral amygdala activation (i.e. attenuation due to reappraisal) was observed, along with attenuation of right insula. Such bilateral amygdala activation was also observed for distancing separately (i.e. Distancing > Null), but not for reinterpretation separately; no results were significant for Reinterpretation > Null (Table 3).

**Table 3.** Results of meta-analysis for contrast Look Negative > Reappraise Negative

Comparison	Region	MNI coordinates (x, y, z)	Number of voxels	*z* (peak)	Max proportion differential	Mean proportion differential
Distancing + Reinterpretation (combined)	Left amygdala	−24, −3, −18	54	3.72	0.281658	0.211484
	Right amygdala	24, −3, −15	51	3.72	0.270365	0.209166
	Right insula	51, −21, 18	1	3.72	0.178016	0.178016
Distancing > Null	Left amygdala	−24, −3, −18	77	3.72	0.468148	0.302092
	Right amygdala	27, 0, −18	46	3.72	0.368116	0.277267
Reinterpretation > Null	None					
Distancing > Reinterpretation	Left amygdala	−24, −3, −18	56	3.72	0.429148	0.292164
	Right amygdala	27, 0, −18	16	3.72	0.291240	0.238027
Reinterpretation > Distancing	Right orbitofrontal cortex (BA 11)	3, 63, −18	29	3.72	0.265972	0.240626
	Right paracentral lobule (BA 5)	12, −30, 51	6	3.24	0.264338	0.234085
	Right inferior frontal gyrus (BA 47)	18, 30, −9	6	3.54	0.260900	0.230895
	Right superior temporal gyrus (BA 38)	36, 21, −30	6	3.16	0.260900	0.230786
	Right parahippocampal gyrus	30, −45, −9	6	3.72	0.259341	0.235038
	Right superior frontal gyrus (BA 10)	6, 72, 0	9	3.72	0.256935	0.226225
	Right subgenual cingulate cortex (BA 25)	3, 24, −21	6	3.16	0.256424	0.234088
	Right middle temporal gyrus (BA 21)	66, −3, −15	6	3.72	0.256044	0.233016
	Right DLPFC (BA 46)	45, 33, 12	6	3.24	0.255924	0.232910
	Right caudate	3, 15, −6	4	3.54	0.248026	0.226522
	Right precentral gyrus (BA 6)	33, −9, 69	4	3.72	0.242557	0.227300
	Left middle occipital gyrus (BA 19)	−33, −81, 21	7	3.72	0.242394	0.226065
	Right superior temporal gyrus (BA 41)	51, −21, 15	3	3.16	0.238129	0.222337
	Right putamen	30, −15, −15	1	3.24	0.216385	0.216385

BA = Brodmann Area. Proportion Differential = Difference in activation proportion between conditions in brain region.

**Fig. 4.**
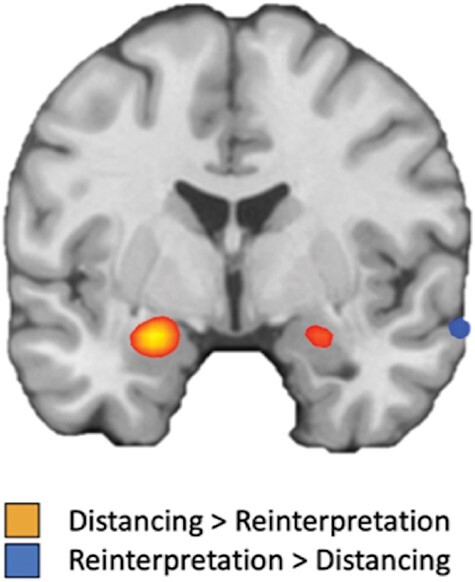
Bilateral amygdala activation for distancing relative to reinterpretation for the Look Negative > Reappraise Negative contrast, reflective of greater prevalence of amygdala attenuation during reappraisal for distancing studies relative to reinterpretation studies.

Finally, we directly contrasted distancing and reinterpretation for the Look Negative > Reappraise Negative contrast. Reinterpretation relative to distancing was associated with attenuation during reappraisal (i.e. for the Look Negative > Reappraise Negative contrast) in medial orbitofrontal cortex along with other distributed frontal, temporal, parietal and occipital foci shown in Table 3. For distancing relative to reinterpretation, the only significant result was bilateral amygdala, reflective of greater prevalence of amygdala attenuation during reappraisal for distancing studies relative to reinterpretation studies (Figure 4; Table 3).

### Behavioral meta-analysis

Behavioral meta-analysis results are shown in Supplemental Material (Table S1). Pooled effect size estimates indicated significant effects of reappraisal as a whole, distancing in particular, and reinterpretation in particular in diminishing negative (or enhancing positive) self-reported affect relative to naturally viewing negative stimuli (Figure S1). A subsequent direct comparison of effect sizes for distancing and reinterpretation did not yield significant differences (Table S2).

## Discussion

In the present meta-analysis, we aimed to unpack the neural mechanisms underlying cognitive reappraisal by investigating the degree of overlap and differentiation in the neural correlates of two principal tactics through which reappraisal can be implemented, psychological distancing and reinterpretation (McRae *et al.*, 2012; Webb *et al.*, 2012; Denny and Ochsner, 2014). To our knowledge, the present study included the largest number of contrasts for distancing and reinterpretation to date in a meta-analysis involving a direct comparison between them. We were further interested in particular in the objective, impartial tactic for implementing psychological distancing (Powers and LaBar, 2019; Shahane and Denny, 2019; Denny, 2020; Shahane *et al.*, 2021). When collapsing across distancing and reinterpretation implementation, for the reappraisal implementation contrast (i.e. Reappraise Negative > Look Negative), we found evidence consistent with that of several prior meta-analyses that have examined reappraisal as a whole, namely engagement of domain-general cognitive control regions—including bilateral DLPFC, left VLPFC, posterior DMPFC/pre-SMA and posterior parietal cortex—along with left posterior temporal cortex (Kalisch, 2009; Buhle *et al.*, 2014; Kohn *et al.*, 2014; Messina *et al.*, 2015; Morawetz *et al.*, 2017).

We then performed meta-analyses examining distancing and reinterpretation separately. For distancing alone, we observed consistent activation in right DLPFC, posterior DMPFC/pre-SMA, bilateral posterior parietal cortex and posterior cingulate cortex. By far the largest significant cluster was located in right DLPFC, with the next largest cluster located in left posterior parietal cortex. These results, particularly including right lateralization for the DLPFC result as well as all other regions found above including pre-SMA, bilateral posterior parietal cortex and posterior cingulate cortex, are consistent with those of a recent meta-analysis by Powers and LaBar for distancing in particular (Powers and LaBar, 2019). For reinterpretation, we observed consistent activation in left VLPFC and left posterior DLPFC/premotor cortex, consistent with a meta-analysis from Messina and colleagues that examined reinterpretation (i.e. reappraisal of stimuli) in particular (Messina *et al.*, 2015).

We then performed a conjunction analysis to assess regions that showed consistent activation patterns for both distancing and reinterpretation. Consistent with prior meta-analyses for reappraisal as a whole, we found common activation in left posterior DLPFC/premotor cortex, posterior DMPFC/pre-SMA and left posterior temporal cortex (Kalisch, 2009; Buhle *et al.*, 2014; Kohn *et al.*, 2014; Messina *et al.*, 2015; Morawetz *et al.*, 2017). Notably, while we did not observe left posterior temporal cortex activity in meta-analyses for distancing and reinterpretation separately, we did observe consistent activation in this middle temporal gyrus region in the conjunction analysis across distancing and reinterpretation, and furthermore did not observe activity in VMPFC, consistent with neurocognitive models for reappraisal that include a role for lateral temporal cortex in engaging semantic and perceptual representations in ways that indirectly modulate emotional responses (Ochsner *et al.*, 2012; Buhle *et al.*, 2014; Powers and LaBar, 2019).

Finally, we directly compared the neural correlates of distancing and reinterpretation. We found that distancing resulted in significantly more consistent activation in right DLPFC and left posterior parietal cortex relative to reinterpretation, whereas implementing reinterpretation was associated with significantly more consistent activation in left VLPFC relative to distancing. These results are consistent with recent meta-analyses examining distancing (Powers and LaBar, 2019) and reinterpretation in isolation (Messina *et al.*, 2015). However, to our knowledge, the present results represent the most comprehensive direct comparison of these reappraisal tactics to date. While two prior meta-analyses have directly compared the neural correlates of distancing and reinterpretation (Messina *et al.*, 2015; Picó-Pérez *et al.*, 2017), the present meta-analysis utilized more than double the number of contrasts for both distancing and reinterpretation than has been previously reported in these direct comparisons.

In addition, we examined the reverse contrast (i.e. Look Negative > Reappraise Negative), reflective of regions that have shown significant attenuation in activity due to reappraisal. For distancing, consistent bilateral amygdala attenuation was observed along with attenuation of right insula, consistent with prior work on regions modulated by reappraisal (Ochsner and Gross, 2005, 2008; Ochsner *et al.*, 2012; Buhle *et al.*, 2014; Denny and Ochsner, 2018). A direct contrast of distancing and reinterpretation for this contrast revealed bilateral amygdala activation, reflective of a greater prevalence of amygdala attenuation during reappraisal for distancing relative to reinterpretation studies. This result is consistent with recent work directly contrasting the neural correlates of distancing and reinterpretation in the same participants (Hermann *et al.*, 2021).

### Behavioral meta-analysis

Further, in a behavioral meta-analysis, we found that both distancing and reinterpretation led to significant reductions in self-reported negative affect (or increases in self-reported positive affect) relative to unregulated baseline conditions. The pooled behavioral effect size estimate for distancing was non-significantly larger and more heterogeneous, relative to the estimate for reinterpretation; this should be interpreted with caution, and the observed heterogeneity may reflect important differences in operationalization of each tactic across studies. Broadly, these results are consistent with those of (Webb *et al*., 2012), who showed in a behavioral meta-analysis of emotion regulation strategies that reappraisal-by-perspective taking—akin to distancing—yielded a relatively large effect size estimate, although not significantly larger than reappraising the emotional stimulus—akin to reinterpretation. The present results are also broadly consistent with those of prior work that has directly compared behavioral effects of longitudinal reappraisal training using distancing and reinterpretation separately, with evidence for lower self-reported negative affect, and unique reductions in perceived stress, for distancing (Denny and Ochsner, 2014). Future behavioral meta-analyses may continue to probe this direct comparison.

### Implications of present results for neural models of distancing and reinterpretation

The present results extend psychological and neurocognitive models for distancing and reinterpretation as specific reappraisal tactics. Both distancing and reinterpretation are broadly associated in the present results with posterior DMPFC/pre-SMA, DLPFC and VLPFC activity that may reflect mentalizing, holding reappraisals in working memory and selecting appropriate reappraisals and inhibiting goal-irrelevant information, respectively, consistent with prior work (Ochsner and Gross, 2005, 2008; Denny *et al.*, 2012; Ochsner *et al.*, 2012; Buhle *et al.*, 2014; Denny and Ochsner, 2018). However, the present results provide support for the right laterality effect in DLPFC activity for distancing even relative to reinterpretation, consistent with a recent meta-analysis for distancing in isolation from Powers and LaBar (2019). This may reflect increased engagement of working memory processes for distancing in particular relative to reinterpretation.

Further, activity in left posterior parietal cortex/TPJ, which has been previously associated with convergent processing of mentalizing, attention, language and memory (Carter and Huettel, 2013), was more robustly activated for distancing compared to reinterpretation, consistent with an emerging perspective that posits that distancing involves managing multiple perspectives and mental states during reappraisal (Powers and LaBar, 2019). Additionally, the present parietal cortex results associated with distancing are consistent with distancing’s key role in mindfulness-based training, particularly regarding decentering (Kross and Ayduk, 2017; Hanley *et al.*, 2021), as well as prior neuroimaging work showing increases in parietal cortex activity following mindfulness training (Goldin *et al.*, 2013). Overall, these results point toward the conceptual overlap between distancing and mindfulness, which may help unpack mechanisms for mindfulness’ contribution to well-being (Hanley *et al.*, 2021).

In addition, while prior meta-analyses have shown left as well as right VLPFC to be consistently activated during reinterpretation (Messina *et al.*, 2015), the present results suggest that left lateralized VLPFC activity is particularly associated with reinterpretation, which may reflect conceptual specialization for left VLPFC in mnemonic cognitive control mechanisms (Badre and Wagner, 2007) relevant to engaging reappraisal in a relatively situation/stimulus-dependent manner (Denny, 2020).

Finally, the present results suggest that while amygdala attenuation is broadly associated with reappraisal implementation, these effects are most prevalent for distancing relative to reinterpretation studies. This may have implications for the selection and examination of activity patterns in common affect-related regions-of-interest like amygdala for studies that operationalize reappraisal via particular tactics like distancing and reinterpretation. Further, future studies may examine how individual differences in reappraisal usage predict amygdala responsiveness and other neural correlates of reappraisal (Drabant *et al.*, 2009) as well as whether and how individual differences in amygdala attenuation during distancing and reinterpretation may be reflective of vulnerability to psychopathology (Kanske *et al.*, 2015; Rodman *et al.*, 2019).

### Limitations and future directions

The present meta-analysis targeted distancing studies that operationalized distancing via thinking about a negative emotional stimulus as an objective, impartial observer. While this is a key facet of distancing (Powers and LaBar, 2019; Shahane and Denny, 2019; Denny, 2020; Shahane *et al.*, 2021), and while it resulted in near parity with number of studies examining reinterpretation in the present work, future meta-analyses may separably examine the neural mechanisms of spatial, temporal, objective and hypothetical forms of distancing (Trope and Liberman, 2010; Powers and LaBar, 2019) *vs* reinterpretation, although hypothetical distancing is often operationalized as reinterpretation (e.g. Burklund *et al.*, 2014; Silvers *et al.*, 2015). Further, in practice, multiple emotion regulation tactics may be combined (Ford *et al.*, 2019), thus presumably resulting in further overlap in neural mechanisms. Future work may continue to probe these distinctions and patterns of overlap.

Further, the present meta-analysis included small volume corrected results (e.g. for amygdala activity) using thresholds as defined by the authors of the included studies. As these thresholds have shown considerable variance, future work may examine this factor in greater detail.

Finally, the present meta-analysis was also limited to healthy adults. It will be important to examine these questions in additional age groups, including children and adolescents, and among older adults in particular. Further, future individual studies and meta-analyses may make further comparisons to the neural mechanisms supporting particular reappraisal tactics in affectively disordered populations (Picó-Pérez *et al.*, 2017; Zilverstand *et al.*, 2017).

## Conclusions

Building on prior meta-analyses, the present meta-analysis on the overlap and differentiation between two widely studied tactics for cognitive reappraisal, psychological distancing via objective appraisal and reinterpretation, revealed relative specialization for distancing relative to reinterpretation in recruiting right DLPFC and left posterior parietal cortex, whereas reinterpretation relative to distancing showed relative specialization in recruiting left VLPFC. We also showed greater prevalence of amygdala attenuation during reappraisal for distancing relative to reinterpretation studies. Overall, the present results suggest future applications in region-of-interest specification and neural network analysis in studies focusing on specific reappraisal tactics and moreover contribute to an evolving psychological and neurocognitive model for reappraisal and the separable tactics through which it can be implemented.

## Supplementary Material

nsad050_Supp

## Data Availability

The meta-analytic database is available via: https://osf.io/68p54/?view_only=0a7f4b46c26d4ad38569808f06b4b990.
