# Supplementary material for: Unpacking reappraisal: a systematic review of fMRI studies of distancing and reinterpretation
Source: Soc Cogn Affect Neurosci. 2023 Sep 27;18(1):nsad050. doi: 10.1093/scan/nsad050 (PMC10561539; doi:10.1093/scan/nsad050)
Supplement: nsad050_Supp [file nsad050_supp.zip › scan-22-114-File009.pdf]

Supplemental Methods

fMRI Meta-Analysis

Quality Assessment

Methodological quality assessments of the included studies were conducted using Version 2 of the Cochrane risk-of-bias tool (https://methods.cochrane.org/bias/resources/rob-2-revised-cochrane-risk-bias-tool-randomized-trials). This tool assesses risk of bias by examining five domains of bias: 1) the randomization process, 2) deviations from intended interventions, 3) missing outcome data, 4) measurement of the outcome, 5) selection of the reported result, and overall risk of bias. Each domain can be categorized/scored as having high levels, low levels, or some concerns of bias. Risk of bias was assessed independently by three trained researchers; any disagreements were resolved via discussion (see Denny_et_al_Reapp_Meta_Analysis_ROB.xlsx included as Supplementary Material). Because the type of studies selected for this meta-analysis included experimental designs where participants received all conditions, risk of bias due to randomization, deviations from intended interventions, and measurement of outcome for the included studies was low.

Heterogeneity was assessed via quality assessments (see Denny_et_al_Reapp_Meta_Analysis_ROB.xlsx included as Supplementary Material). We addressed the robustness of synthesized results by using all results (i.e., individual study foci) that pertained to contrasts of interest that met multiple comparison correction as defined by the authors of each study. All multiple comparison corrected results from individual studies, excluding local maxima and including small volume corrected results,

were included. Thus, any risk of bias due to missing data was low. This meta-analysis was not preregistered.

Behavioral Meta-Analysis

Study coding and Data Collection

To complement our neuroimaging meta-analysis of fMRI-based studies of distancing and reinterpretation, we performed a meta-analysis comparing behavioral effects of reappraisal implementation using either distancing or reinterpretation among the studies included in the neuroimaging meta-analysis. Cohen's *d* effect size estimates and its corresponding standard error was calculated from the means and standard deviations of affect ratings obtained from the Reappraise Negative (i.e., either distancing or reinterpretation) and Look Negative conditions across studies. These data were derived from the papers themselves, provided by the authors via email, or obtained from raw data provided by the authors. Any study from which we could not collect data on the means or standard deviations for both conditions was excluded from analyses (e.g., the authors we reached out to either did not have access to that information or did not respond to our inquiries).

Due to the diverse scales used in the studies included in our analyses, 3 coders determined whether each effect size was in the expected direction (i.e., less negative or more positive affect in the Reappraise vs. Look condition, in accordance with the authors' hypothesis and our own). There were no disagreements during coding, and any uncertainties about the language of the scale were resolved either through coder collaboration and by consulting with the principal investigator. The absolute value of

effect sizes for studies in which the expected effect was observed was calculated. Only one study showed an effect in the opposite direction (i.e., opposite to the authors' hypothesis and our own) and thus was coded as a negative effect size (see Table S1). Further, one study (Dorfel et al., 2014) contained separate comparisons for distancing and reinterpretation, respectively, versus the Look condition and thus contributes two effects to the meta-analysis (Table S1).

Data Analysis

All analyses were conducted in RStudio, version 4.2.1 (R Core Team, 2022). Meta-analyses of within-group mean differences in R require pre-calculated effect sizes and standard errors, so we first calculated those values as described above. We then used the metagen() function from the *meta* package in R to find the pooled effect sizes for (1) all studies comparing Reappraise Negative to Look Negative (i.e., collapsing across distancing versus reinterpretation); (2) only studies which compared distancing to a look condition; and (3) only studies which compared reinterpretation to a look condition. We modeled heterogeneity with random effects models for all meta-analyses, with the Restricted Maximum Likelihood procedure as our variance estimator (Viechtbauer, 2005) and the Knapp-Hartung adjustment to control for uncertainty in between-study heterogeneity, to reduce chances of false positives (Knapp & Hartung, 2003; Langan et al., 2019).

The output from the metagen() function provides the pooled mean effect sizes and associated 95% confidence intervals, *p*-values, tau-values (SD of the random effects), and other information. To assess differences in the pooled effect sizes of the distancing vs. look studies and the reinterpretation vs. look studies, we conducted a subsequent meta-

regression with a categorical moderator (i.e., distancing or reinterpretation) using the rma() function from the *metafor* package in R.

Supplemental Results

Behavioral Meta-Analysis

Study Selection and Study Characteristics

A total of 21 studies were included in our behavioral meta-analysis, yielding a combined 22 different effect sizes (as one study investigated both strategies; Dorfel et al., 2014) for distancing (k = 8) and reinterpretation (k = 14). Characteristics of the included studies can be found in Table S1.

Reappraisal Overall

The pooled effect size of Reappraisal vs. Look as a whole was statistically significant (d = 1.15, t = 5.61, p <.001, 95% CI [.72, 1.57]), indicating a significant effect of reappraisal overall in diminishing negative (or enhancing positive) self-reported affect relative to naturally viewing negative stimuli. Further, a test of between-study heterogeneity revealed that our tau-squared statistic is significantly greater than zero ($\tau 2$ = .60, 95% CI [.40, 2.20]), indicating that between-study heterogeneity exists in the data and justifies the decision to use a random-effects model.

Distancing

The pooled effect size of Distancing vs. Look was statistically significant (d = 1.58, t = 2.83, p =.026, 95% CI [.26, 2.91]), indicating a significant effect of distancing in particular in diminishing negative (or enhancing positive) self-reported affect relative to naturally viewing negative stimuli. As for results for Reappraisal vs. Look, a test of between-study heterogeneity revealed that our tau-squared statistic is significantly greater

than zero ($\tau 2$ = 2.07, 95% CI [.81, 11.37]), indicating that between-study heterogeneity exists in the data and justifies the decision to use a random-effects model. A forest plot for distancing study results for the behavioral meta-analysis is shown in Figure S1A.

Reinterpretation

The pooled effect size of Reinterpretation vs. Look was statistically significant (*d* = 0.95, *t* = 6.07, *p* <.001, 95% CI [.61, 1.29]), indicating a significant effect of reinterpretation in particular in diminishing negative (or enhancing positive) self-reported affect relative to naturally viewing negative stimuli. As with the previous analyses, a test of between-study heterogeneity revealed that our tau-squared statistic is significantly greater than zero ($\tau 2$ = .26, 95% CI [.10, .85]), indicating that between-study heterogeneity exists in the data and justifies the decision to use a random-effects model. A forest plot for reinterpretation study results for the behavioral meta-analysis is shown in Figure S1B.

Comparing Behavioral Effect Sizes for Distancing versus Reinterpretation

To determine if the difference in effect sizes between distancing and reinterpretation is significant, we first compared the 95% confidence intervals to each other and found that they overlap, meaning we are unable to determine from the current analyses whether the difference is significant. Thus, we conducted a two-group moderator analysis using the rma() function in R, with distancing as one group and reinterpretation as the other. Results from this analysis can be found in Table S2. The omnibus test from this analysis was not statistically significant (Q_M = 1.67, df = 1, *p* =.196), indicating that there is not sufficient evidence to conclude that there is a significant difference in effect sizes between distancing and reinterpretation.

Supplementary Tables

Table S1. Studies included in the behavioral meta-analysis

Author	Healthy Sample N	Cognitive Reappraisal Strategy Used	Cohen's *d* Behavioral Effect Size (SE)
Burklund et al., 2014	39	Reinterpretation	0.98 (.203)
Denny et al., 2015a	21	Distancing	1.01 (.290)
Denny et al., 2015b	17	Distancing	1.37 (.378)
Dorfel et al., 2014	17	Distancing;	0.44 (.274)
	19	Reinterpretation	0.61 (.269)
Gianaros et al., 2014	157	Reinterpretation	1.03 (.100)
McRae et al., 2010	18	Reinterpretation	1.92 (.449)
Modinos et al., 2010	18	Reinterpretation	2.00 (.461)
Nelson et al., 2015	21	Reinterpretation	1.08 (.298)
Opitz et al., 2012	31	Reinterpretation	-0.08 (.186)
Paschke et al., 2016	108	Distancing	1.72 (.154)
Reinecke et al., 2015	18	Reinterpretation	1.52 (.387)
Schulze et al., 2011	15	Distancing	0.15 (.281)
Seidel et al., 2018	36	Distancing	0.96 (.210)
Shermohammed et al., 2017	54	Reinterpretation	1.23 (.186)
Simsek et al., 2017	13	Reinterpretation	1.51 (.476)
Sripada et al., 2014	49	Reinterpretation	0.57 (.158)
Townsend et al., 2013	26	Reinterpretation	0.86 (.243)
Vanderhasselt et al., 2013	42	Reinterpretation	0.10 (.159)
Winecoff et al., 2013	31	Distancing	5.50 (.782)
Xie et al., 2016	19	Distancing	2.36 (.503)
Ziv et al., 2013	27	Reinterpretation	1.13 (.262)

Table S2. Behavioral Effects Two-Group Moderator Analysis

	Estimate	*SE*	*z*	*p*	95% CI
Intercept	1.45	.31	4.75	.000	[.85, 2.05]
StrategyR	-.49	.38	-1.29	.196	[-1.23, .25]

Note. StrategyR = difference of Reinterpretation group from the Distancing group, which is the reference group.

Supplementary Figures

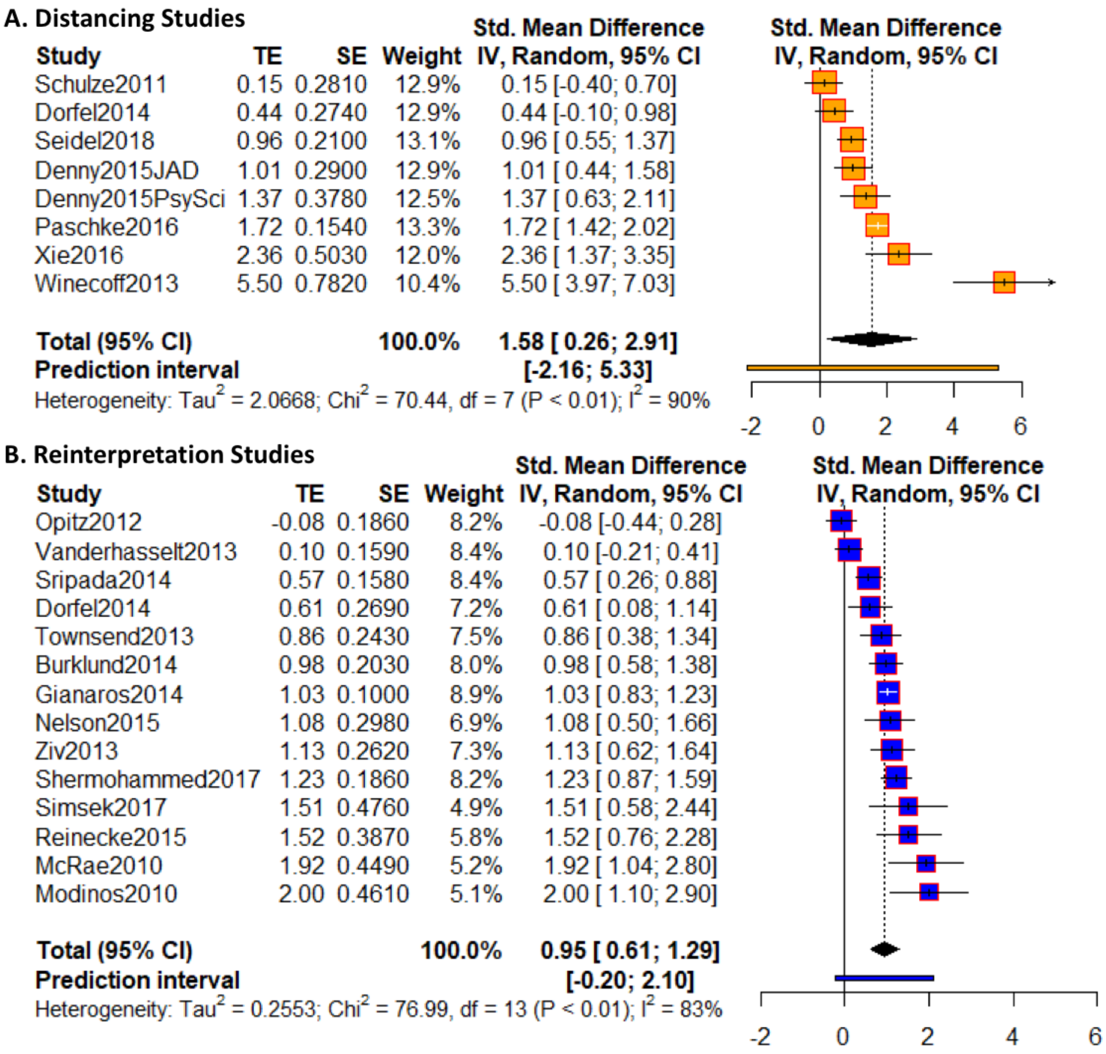

Figure S1. Behavioral meta-analysis results. (A) Distancing studies. (B). Reinterpretation studies.

References

Knapp, G. & Hartung, J. (2003). Improved tests for a random effects meta-regression with a single covariate.” *Statistics in Medicine*, *22*(17), 2693–2710.

Langan, D., Higgins, J.P.T., Jackson, D., Bowden, J., Veroniki, A.A., Kontopantelis, E., Viechtbauer, W., & Simmonds, M. (2019). A comparison of heterogeneity variance estimators in simulated random-effects meta-analyses. *Research Synthesis Methods, 10*(1), 83–98.

R Core Team (2022). R: A language and environment for statistical computing. *R Foundation for Statistical Computing*. Vienna, Austria. https://www.R-project.org/.

Viechtbauer, W. (2005). Bias and efficiency of meta-analytic variance estimators in the random-effects model. *Journal of Educational and Behavioral Statistics, 30*(3), 261–93.
